# Serum-based diagnosis of *Pneumocystis* pneumonia by detection of *Pneumocystis jirovecii* DNA and 1,3-β-D-glucan in HIV-infected patients: a retrospective case control study

**DOI:** 10.1186/s12879-019-4289-4

**Published:** 2019-07-23

**Authors:** Helena Hammarström, Anna Grankvist, Isabell Broman, Nahid Kondori, Christine Wennerås, Magnus Gisslen, Vanda Friman

**Affiliations:** 1 Department of Infectious Diseases, Institute of Biomedicine, Sahlgrenska Academy, University of Gothenburg, Gothenburg, Sweden; 2 Region Västra Götaland, Sahlgrenska University Hospital, Department of Infectious Diseases, Gothenburg, Sweden

**Keywords:** *Pneumocystis jirovecii*, *Pneumocystis* pneumonia, HIV, AIDS, Diagnosis, Polymerase chain reaction, 1,3-beta-d-glucan

## Abstract

**Background:**

*Pneumocystis jirovecii* pneumonia (PCP) is one of the most common HIV-related opportunistic infections. The diagnosis of PCP is based on analyses from respiratory tract specimens which may require the invasive procedure of a diagnostic bronchoscopy. The objective of this study was to evaluate the diagnostic potential of *Pneumocystis jirovecii* PCR in serum combined with the 1,3-β-D-glucan (betaglucan) test for the diagnosis of PCP in HIV-infected patients.

**Methods:**

This was a retrospective case-control study including serum samples from 26 HIV-infected patients with PCP collected within 5 days prior to the start of PCP treatment, 21 HIV-infected control subjects matched by blood CD4^+^ cell counts, and 18 blood donors. The serum samples were analyzed for *Pneumocystis jirovecii* PCR and betaglucan. The reference standard for PCP was based on previously described microbiological and clinical criteria.

**Results:**

All patients with PCP had detectabe *Pneumocystis jirovecii* DNA in serum yielding a sensitivity for the *Pneumocystis jirovecii* PCR assay in serum of 100%. All blood donors had negative *Pneumocystis* PCR in serum. The specificity when testing HIV-infected patients was 71%, but with a PCR Cycle threshold (Ct) value of 34 as cut-off the specificity was 90%. At a putative pretest probaility of 20%, the negative and positive predictive value for the *Pneumocystis* PCR assay in serum was 0.99 and 0.71, respectively. Betaglucan with cut-off level 200 pg/ml combined with a positive *Pneumocystis jirovecii* PCR result had sensitivity and specificity of 92 and 90%, respectively. The concentration of *Pneumocystis jirovecii* DNA in serum samples, expressed by the PCR Ct values, correlated inversely to the betaglucan levels in serum.

**Conclusion:**

In this case-control study including 70% of all HIV-infected patients with PCP treated at Sahlgrenska University Hospital during a time period of 13 years, *Pneumocystis* PCR analysis on serum samples had a very high sensitivity and negative predictive value for the diagnosis of PCP in HIV-infected patients. A serum-based diagnostic procedure either based on *Pneumocystis jirovecii* PCR alone or in combination with betaglucan analysis may thus be feasible and would facilitate the care of HIV-infected patients with suspected PCP.

## Background

Pneumonia caused by the fungus *Pneumocystis jirovecii* (*Pj)* is one of the most common HIV-related opportunistic infections [1]. The diagnosis of Pneumocystis pneumonia (PCP) formerly relied solely on immunofluorescent staining and microscopic examination of the microorganism in samples from the lower respiratory tract, but polymerase chain reaction (PCR) methodology has been found to be more sensitive for the detection of *Pj* in respiratory specimens [2]. *Pj* DNA can also be detected by PCR in respiratory tract specimens from patients without clinical signs of pneumonia [3, 4], and *Pj* is now considered to be a frequent colonizer of the respiratory tract in patients with HIV [5–7].

*Pj* primarily resides in the alveoli of the lungs [8], and the concentration of the fungus is higher in specimens from the lower respiratory tract such as induced sputum and bronchoalveolar lavage (BAL) fluid than in upper respiratory tract specimens [9–11]. Consequently, induced sputum and BAL fluid are the recommended specimens for microbiological *Pj* analyses [12]. However, HIV-infected patients with PCP typically present with subacute onset of fever, dyspnoea and non-productive cough [13], and sputum samples may be difficult to obtain. The invasive procedure of a diagnostic bronchoscopy may thus be the only alternative to obtain respiratory specimens from HIV-infected patients for PCP diagnosis.

Previous studies have looked upon the possibility to use blood sample-based analyses for the microbiological diagnosis of PCP. In the 1990s some investigators using conventional PCR reported that *Pj* PCR performed on blood samples had too low sensitivity [2, 14–18]. Recent studies employing real-time PCR methodology show somewhat more promising results [9, 19, 20]. However, these studies lack well-defined clinical categorization of PCP versus *Pj* colonization and include mixed patient groups with various types of underlying immunosuppressive conditions making the results difficult to interpret in the context of PCP in HIV-infected patients.

1,3-ß-D-glucan (betaglucan), a polysaccharide that constitutes the major cell wall component of *Pneumocystis jirovecii*, can be detected in the serum of patients with PCP, and several studies have reported a good diagnostic performance in HIV-infected PCP patients [21–24].

To the best of our knowledge, there are no studies evaluating the diagnostic potential of combining blood-based analyses of *Pj* DNA and betaglucan for the microbiological diagnosis of PCP in HIV-infected patients. The aim of this study was to evaluate whether *Pj* PCR and betaglucan analysis of blood samples could be a diagnostic alternative to the recommended *Pj* PCR performed on specimens from the lower respiratory tract in HIV-infected patients with PCP.

## Methods

### Patients and control subjects

Patients with HIV infection who had received a diagnosis of PCP at Sahlgrenska University Hospital in Gothenburg, Sweden between 2005 and 2018 were identified through the Swedish National Registry for HIV-care (InfCare HIV) [25]. The patients were included in the study provided that they had a serum sample frozen at − 80 °C that had been collected within 5 days prior to the start of PCP treatment, and that they complied with the reference standard for PCP in this study, which was based on the following previously described criteria: *Pj* detected by PCR and/or microscopy with immunofluorescent staining in a sputum or BAL specimen; a minimum of two out of three clinical criteria of PCP (respiratory symptoms consisting of cough and/or dyspnoea, hypoxia, and typical radiological picture such as ground glass opacity on a chest computer tomography scan or diffuse interstitial opacity on chest x-ray); receipt of a full course of treatment for PCP; and no other alternative aetiology to the clinical picture [26]. Compliance with the reference standard for PCP was independently assessed by two infectious disease specialists (H.H and M.G) prior to the analysis of the microbiological tests being evaluated (i.e. *Pj* PCR and betaglucan on serum samples). The severity of PCP was assessed based on the degree of hypoxemia (PaO2/FiO2 index) within the first four days after start of PCP treatment and was categorized as mild (> 200 mmHg) or severe (≤200 mmHg) pneumonia [27, 28].

Twenty-one HIV-infected patients matched for blood CD4^+^ cell counts who were admitted to the Department of Infectious Diseases at Sahlgrenska University Hospital during the same time period and who had not received a clinical diagnosis of PCP were identified through the Swedish National Registry for HIV-care and randomly selected as negative control subjects provided that they had a serum sample collected at the time of HIV diagnosis similarly stored at − 80 °C. Furthermore, serum samples from 18 blood donors were included as additional negative controls.

### Blood samples

Serum samples stored at − 80 °C from the patients with HIV and fresh serum samples from consecutive blood donors were analysed for betaglucan and *Pj* PCR at the Department of Clinical Microbiology at Sahlgrenska University Hospital. All analyses were performed by certified laboratory technicians who were blinded to the clinical data of the patients.

### Betaglucan

The Glucatell® assay kit (Ass. Cape Cod, MA, USA) was used for measuring serum levels of betaglucan by enzymatic reaction. The sera were pretreated and analyzed in duplicate according to the manufacturer’s instructions. A betaglucan level ≥ 80 pg/ml is considered a positive result by the manufacturer, although several studies propose that a higher cut-off level may be preferable [21, 29]. Samples yielding betaglucan values above the maximum level of detection of the assay kit (> 400 pg/ml), were diluted 1:5 and reanalysed to yield a maximum detection limit of 2000 pg/ml.

### *Pneumocystis jirovecii* real time PCR

The real time PCR was performed using the BD MAX open mode System (BD Diagnostics, Franklin Lakes, New Jersey, US), which allows fully automated lysis of samples, DNA extraction and real-time PCR. The primers and probes targeting the mitochondrial gene coding for the large ribosomal subunit (mtLSU) of *Pj* previously described by Dini et al [30] were used with slight modifications: primers F (5′-AAA TAA ATA ATC AGA CTA TGT GCG ATA AGG-3′) and R (5′-GGG AGC TTT AAT TAC TGT TCT GGG-3′); and Taq probe 5′-FAM-AGA TAG TCG AAA GGG AAA C- MGB-3′. A total volume of 500 μL of the sample was transferred into the BD MAX DNA-1 extraction kit (BD Diagnostic) Sample Buffer Tube, which contains a specimen processing control (SPC) that act as a control for extraction and PCR inhibition by the specimen. The PCR master mix was distributed in two different tubes snapped into the BD MAX extraction reagent strip. The first tube was the lyophilized BD MAX DNA MMK (SPC) reagents mix (BD Diagnostics), a universal master mix that also incorporates SPC primer and probe. The second master mix tube was prepared in-house and contained primers and probe (1.6 μM of each primer and 0.8 μM of the probe), 2 μL of primer diluent (provided by BD Diagnostics) and water to a final volume of 12.5 μL. Primers and probe mix were prepared and added to snap-in tubes at the start of the run. Cycling conditions were as follows: 98 °C for 600 s and then 46 cycles of 96 °C for 9 s and 62 °C for 30.7 s. A threshold of 100 units in endpoint fluorescence value was set to determine positive amplification together with approved SPC curve. Samples with non-approved SPC were re-analyzed once after dilution 1:2 in phosphate buffered saline. Prior to this study, 18 frozen concomitant plasma and serum samples from PCP patients and control patients with HIV were analyzed for *Pj* PCR in order to assess the most suitable blood fraction specimen for this PCR assay. In 72% of analyzed plasma samples, the specimen processing control of the PCR analysis was initially negative due to inhibiting factors in the plasma samples yielding inconclusive PCR results. The inhibition of the PCR analysis was overcome by a 1:2 dilution of the plasma samples. The analysis of serum samples yielded conclusive PCR results in the first round of analysis in 100% of the samples. Consequently, serum was considered the most suitable blood fraction for *Pj* PCR analysis.

### Statistics

Comparison of proportions and comparison of medians were calculated by the Fisher’s exact test and Mann Whitney U-test respectively. Sensitivity and specificity was calculated by cross tabulation with 95% confidence interval (CI). Predictive values were calculated by Bayes formula based on a pre-test probability of 20%, consistent with the reported prevalence of PCP among HIV-infected patients with lung infection [31]. Correlation between Betaglucan and PCR cycle threshold (Ct) values was assessed by using Spearman’s correlation coefficient. GraphPad Prism 7.02 software (GraphPad Software Inc.) was used for statistical calculations. A *p* value of < 0.05 was considered statistically significant.

## Results

A total of 37 HIV-infected patients with a clinical diagnosis of PCP were identified by the Swedish National Registry for HIV-care. Eleven patients that lacked a stored serum sample collected prior to the start of PCP treatment (*n* = 7) or that did not comply with the reference standard for PCP (*n* = 4) were excluded. This resulted in 26 patients with PCP eligible for the study. The study population thus included 70% of all HIV-infected patients with PCP treated at Sahlgrenska University Hospital during a time period of 13 years. All 26 patients had positive *Pj* PCR in sputum and/or BAL fluid, 9/26 patients also had microscopic examination with immunofluorescent staining performed where 8/9 had a positive microscopy result. The median duration of respiratory symptoms prior to the collection of the stored serum samples was 28 days (range 4–75 days). All patients received treatment for PCP. Fourteen patients were classified as having a mild PCP and 12 patients had a severe PCP, out of which two patients died as a consequence of PCP. Twenty-four patients (92%) had a favourable outcome.

The medical charts of the 21 HIV-infected control subjects registered in the Swedish National Registry for HIV-care as patients without PCP were reviewed to assure that no patients with PCP were erroneously included. No patients had a clinical diagnosis of PCP, but seven patients had respiratory symptoms. Three of these patients had radiological findings on chest x-ray out of which one was diagnosed with tuberculosis. None of the patients had received trimethoprim–sulfamethoxazole in a therapeutic dose, but four patients received trimethoprim–sulfamethoxazole as PCP prophylaxis. A complete clinical assessment was not permitted due to the retrospective nature of the study; however, based on the available clinical data, none complied with the reference standard for PCP of this study. Consequently, and in order to avoid the risk of exclusion bias, these seven patients remained in the study despite the somewhat uncertain nature of the respiratory symptoms. The remaining 14 control patients had no respiratory symptoms nor clinical signs of opportunistic lung infection.

Background data and results of betaglucan and *Pj* PCR assays performed on serum samples from the HIV-infected patients with PCP, HIV-infected control subjects and blood donors are shown in Table 1. All PCP patients had *Pj* DNA in serum detected by PCR. None of the blood donors were positive by *Pj* PCR in serum, while 6/21 HIV-infected control patients (29%) had *Pj* DNA in serum detectable by PCR. In total, 24/25 patients with PCP (96%) had positive betaglucan in serum (≥80 pg/ml) and 23/25 (92%) had betaglucan levels ≥200 pg/ml. None of the blood donors had detectable betaglucan in serum.

**Table 1:** Patient characteristics and laboratory data

	PCP	no PCP	
	HIV-infected (*n* = 26)	HIV-infected (*n* = 21)	Blood donors (*n* = 18)
Age, yrs., median (range)	47 (25–70)	38 (23–64)	46 (18–59)
Females, n (%)	9 (35)	10 (48)	7 (39)
CD4^+^ cell count, cells/uL, median (range)	35 (0–460)	50 (0–170)	–
Pj PCR in serum			
Patients with positive results, n (%)	26 (100)	6 (29)	0 (0)
Ct value, n, median (range)	29 (25–40)^b^	38 (32–42)	–
BG in serum (pg/ml)			
Patients with BG ≥80	24 (96)^c^	6 (29)	0 (0)
Patients with BG ≥200	23 (92)^c^	3 (14)	0 (0)
BG, median (range)	1328 (50–2000)^c^	50 (50–2000)	50 (50–58)

*PCP Pneumocystis jirovecii* pneumonia, *Ct* cycle threshold, *BG* 1,3-ß-D-glucan

^a^Serum samples collected at hospital admission due to newly diagnosed HIV-infection

^b^The Ct values from three patients with PCP were excluded since their samples were diluted 1:2 prior to analysis

^c^One sample from a patient with PCP was not analyzed for BG due to insufficient volume of serum

The sensitivity and specificity of the *Pj* PCR and betaglucan assays performed on serum samples are shown in Table 2. The *Pj* PCR assay had 100% sensitivity for PCP diagnosis in this study. The specificity was 100% when testing serum from blood donors, but 71% when testing serum samples from HIV-infected control subjects. Receiver operating characteristics curve analysis of PCR Ct values showed that a Ct value of 34 was the optimal cut-off level for this PCR-assay when performed on serum samples. When using a Ct value of ≤34 as cut-off for a positive test result, the specificity increased to 90% with a retained high sensitivity of 96%. Betaglucan analysis of serum samples had a sensitivity > 92% at cut-off levels 80 and 200 pg/ml. The specificity of betaglucan was 100% when testing blood donors but 86% at cut-off level 200 pg/ml when testing the HIV-infected control subjects. When a combination of positive *Pj* PCR and betaglucan ≥200 pg/ml was used as the determinant of a positive test result, the specificity increased to 90%.

**Table 2:** Sensitivity and specificity of *Pj* PCR and betaglucan in serum

Index tests in serum	Sensitivity (%) (95% CI)	Specificity (%) (95% CI)	
	Patients with PCP	HIV-infected control patients	Blood donors
	*n* = 26	*n* = 21	*n* = 18
*Pj* PCR positive	100 (87–100)	71 (50–86)	100 (87–100)
*Pj* PCR with Ct ≤34^a^	96 (79–100)	90 (71–98)	100 (82–100)
BG ≥80 pg/ml^b^	96 (80–100)	71 (50–86)	100 (82–100)
BG ≥200 pg/ml^b^	92 (75–99)	86 (65–95)	100 (82–100)
BG ≥400 pg/ml^b^	88 (70–96)	90 (71–98)	100 (82–100)
*Pj* PCR positive *and* BG ≥200 pg/ml^b^	92 (75–99)	90 (71–98)	100 (82–100)

*Pj Pneumocystis jirovecii, PCP Pneumocystis jirovecii* pneumonia, *CI* confidence interval, *BG* 1,3-ß-D-glucan

^a^Three PCP patients whose serum samples had been analysed after 1:2 dilution were excluded

^b^One PCP patient whose serum sample was not analyzed for BG due to insufficient volume of serum was excluded

The positive and negative predictive values of the *Pj* PCR and betaglucan assays performed on serum samples for the diagnosis of PCP at a putative pre-test probability of 20% [31] are shown in Table 3. The negative predictive values of *Pj* PCR and betaglucan at different cut-off levels for the diagnosis of PCP were very high overall (> 0.97). The highest positive predictive value (0.7) was found for the *Pj* PCR assay with Ct value ≤34 as cut-off for a positive test result or for the combination of a positive *Pj* PCR and a betaglucan level ≥ 200 pg/ml.

**Table 3:** Predictive values of *Pj* PCR and betaglucan in serum at a pre-test probability of 20%

Index tests in serum	Positive predictive value	Negative predictive value
*Pj* PCR positive	0.46	1.0
*Pj* PCR with Ct ≤34^a^	0.71	0.99
BG ≥80 pg/ml^b^	0.45	0.99
BG ≥200 pg/ml^b^	0.63	0.98
BG ≥400 pg/ml^b^	0.69	0.97
*Pj* PCR positive *and* BG ≥200 pg/ml^b^	0.70	0.98

*Pj Pneumocystis jirovecii, PCP Pneumocystis jirovecii* pneumonia, *BG* 1,3-ß-D-glucan

The results are based on 26 patients with PCP and 21 HIV-infected negative controls

^a^Three PCP patients whose serum samples had been analysed after 1:2 dilution were excluded

^b^One PCP patient whose serum sample was not analyzed for BG due to insufficient volume of serum was excluded

As mentioned above, six HIV-infected control patients had detectable *Pj* DNA in serum. Five of these patients belonged to the group of previously described control patients who had respiratory symptoms but who did not comply with the reference standard for PCP. The clinical characteristics of the six HIV-infected control subjects with positive *Pj* PCR results in serum are shown in Table 4. The median Ct value in serum of the 6 PCR positive negative control patients was higher than that of the 26 PCR positive patients with PCP (38 and 29, respectively; *p* < 0.001).

**Table 4:** HIV-infected control patients with positive *Pj* PCR in serum

Pat	CD4 cell count (cells/uL)	Criteria of the PCP reference standard of this study						Outcome	Index tests	
		Respiratory symptoms	Radiologic findings	Hypoxia	*Pj* analysis respiratory specimen	TMP-SMX	Other lung infection		BG (pg/ml)	*Pj* PCR in serum Ct value
1	100	Dry cough	Unknown	Unknown	Unknown	No	No	Survival	> 2000	32
2	30	Dry cough	Discrete interstitial opacity	Unknown	Unknown	Prophylaxis dose	No	Survival	807	33
3	10	Dry cough	Normal	Unknown	Unknown	No^b^	No	Survival	< 50	39
4	80	Dyspnea	Discrete GGO	Unknown	PCR neg^a^	No	No	Survival	60	38
5	50	Productive cough and dyspnea	Normal	No	PCR pos^a^	Prophylaxis dose	No	Survival	177	42
6	50	No	Normal	No	Unknown	No	No	Survival	< 50	39

*PCP Pneumocystis jirovecii* pneumonia, *Pj Pneumocystis jirovecii, TMP-SMX* trimethoprim–sulfamethoxazole, *BG* 1,3-ß-D-glucan, *Ct* Cycle threshold, *GGO* ground glass opacity

For patients where *Pj* analysis, radiological exam or measurement of SpO_2_ was not performed, the data is noted as unknown

^a^PCR on sputum samples

^b^received treatment for toxoplasmosis with pyrimethamine and sulphonamide

**Fig. 1:**
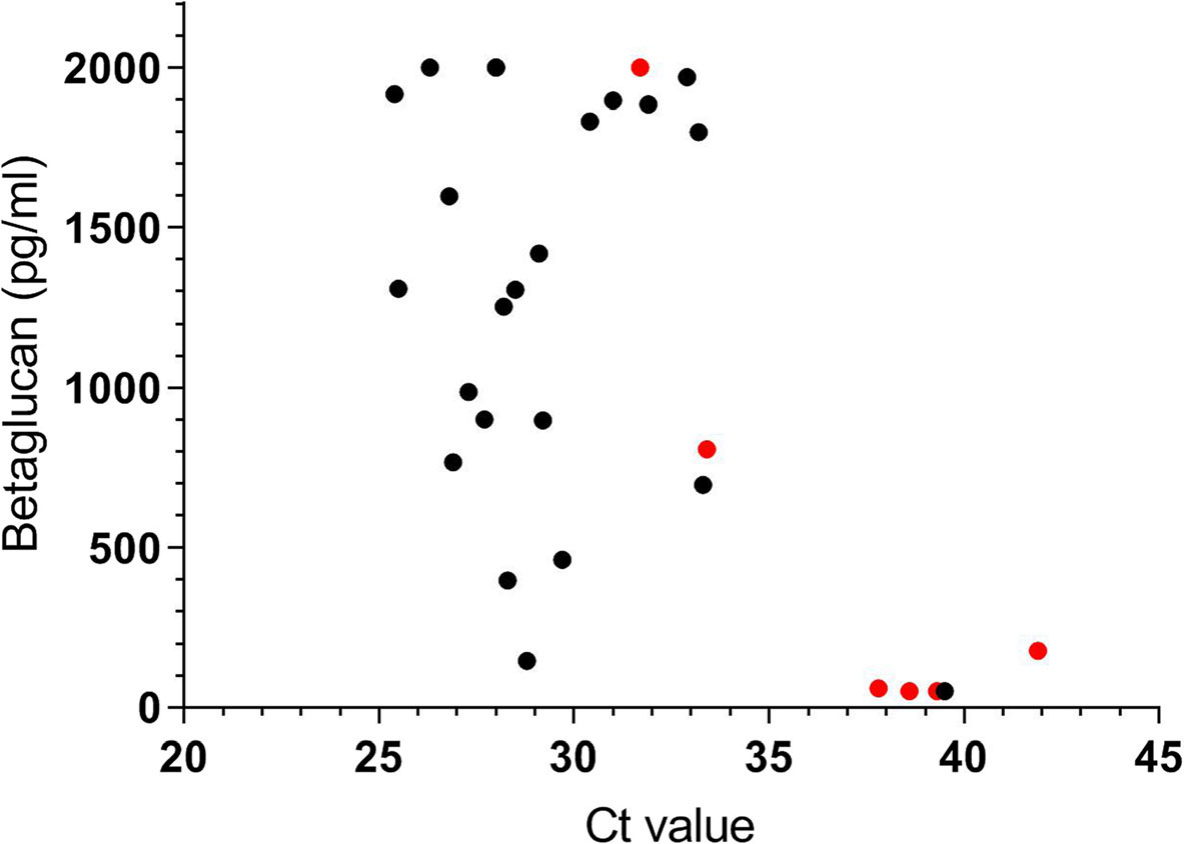
Correlation of Betaglucan and PCR Ct values in serum. Values of betaglucan and PCR Ct in serum from 29 patients with positive *Pj* PCR in serum samples. Black dots represent patients with PCP (*n* = 23), and red dots represent HIV-infected controls (*n* = 6). Three patients with 1:2 diluted serum samples out of which one also had inconclusive BG result were excluded. Correlation was assessed by Spearman’s correlation coefficient: r = − 0.5 (− 0.7 to − 0.1), *p* < 0.01

As shown in Fig. 1, the betaglucan levels tended to be higher in patients with higher concentrations of *Pj* DNA in serum, i.e. patients with lower PCR Ct values (Spearman’s correlation coefficient r = − 0.5). The patients with PCP and the serum PCR-positive control subjects tended to separate into two clusters where the majority of serum PCR-positive control subjects had higher Ct-values and lower betaglucan levels.

**Fig. 2:**
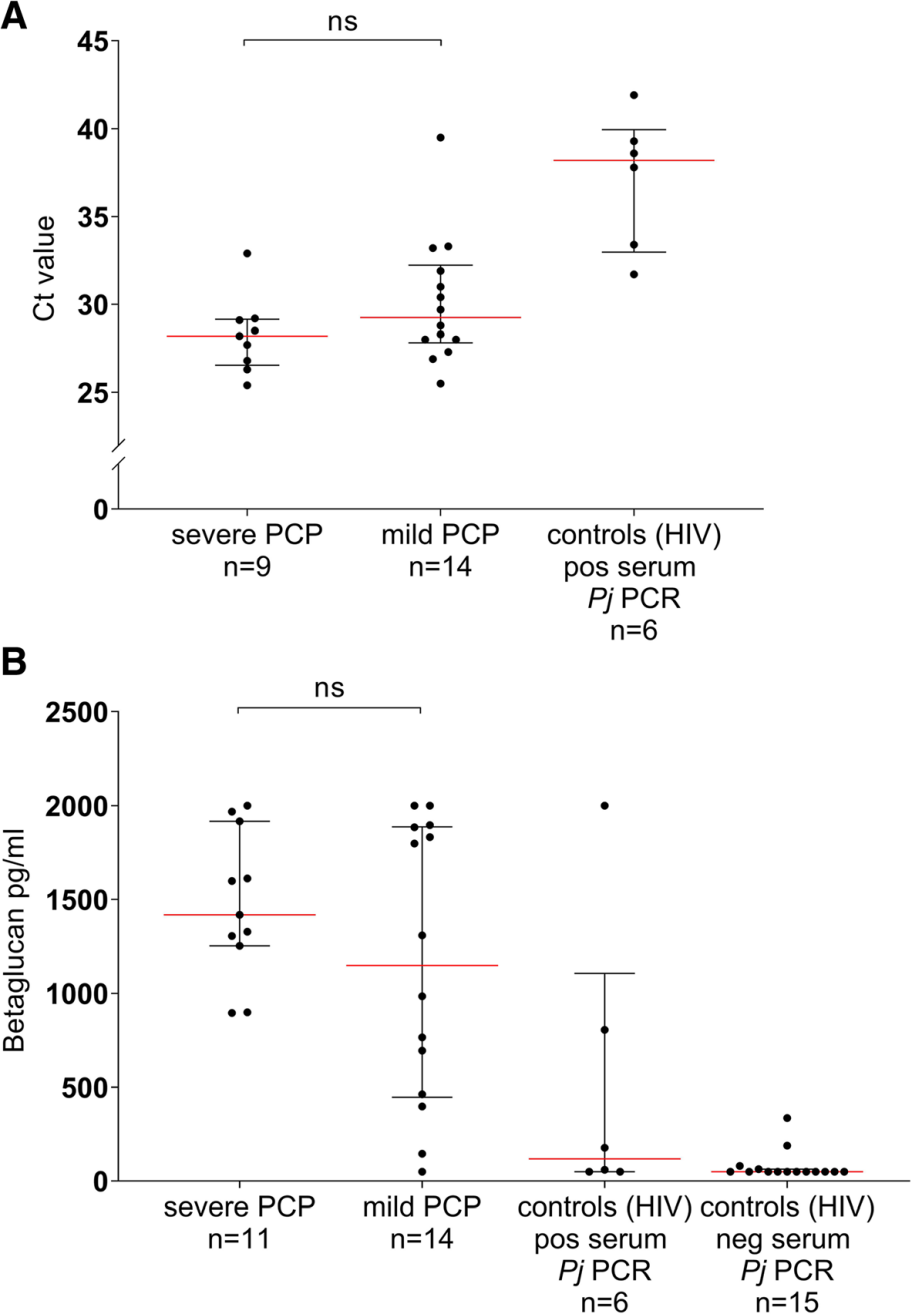
PCR Ct values and Betaglucan levels in serum in patients with severe and mild PCP and in HIV-infected control subjects**.** Ct values (**a**) and Betaglucan levels (**b**) with medians and interquartile ranges in serum samples obtained from patients with severe, mild and no Pneumocystis pneumonia (PCP). Medians were compared by Mann Whitney U-test. Three PCP patients whose serum samples had been analysed after 1:2 dilution (**a**) and one patient whose serum sample was not analyzed for BG due to insufficient volume of serum (**b**) were excluded

PCR Ct values and betaglucan levels in serum in the patients with PCP, categorized into severity of disease, are presented in Fig. 2. There was a difference in median PCR Ct values and betaglucan levels in serum samples between patients with mild or severe PCP, but the difference did not reach statistical significance (Fig. 2).

Three of the patients with PCP did not have a sufficient volume of stored serum for the PCR analysis and the samples were diluted 1:2 with phosphate buffered saline prior to analysis. These three patients were excluded from the analyses that were dependent upon the PCR Cycle threshold (Ct) values. One of them additionally lacked serum for the BG analysis.

## Discussion

This study shows that real time-PCR analysis of *Pj* in serum is a highly sensitive method for PCP diagnosis in patients with HIV. In fact, all patients with PCP included in this study had detectable *Pj* DNA in serum giving a sensitivity of 100%.

Previous studies from the 1990s evaluating the diagnostic potential of PCR for *Pj* using blood samples yielded conflicting results. Two studies evaluating *Pj* PCR performed on serum samples from a group of patients with PCP and various underlying immunosuppressive conditions showed a very low frequency of positivity (10% or less) [14, 18]. Other studies looking at circulating *Pj* DNA exclusively in patients with HIV showed diverging sensitivity estimates ranging from 0 to 86% [15–17, 32]. Nevertheless, these studies all employed conventional PCR methodology with manual DNA extraction with poorer precision and lower sensitivity for DNA detection compared to the automated DNA extraction and real time-PCR methodology used today. A more recent study evaluating concomitant BAL and serum samples from 63 patients with suspected pneumonia and various underlying immunosuppressive disorders showed that 9/10 of patients with PCP and positive microscopic immunofluorescent analysis of BAL samples had circulating *Pj* DNA in serum detected by real time-PCR [19]. In analogy with our study, this suggests that with the more modern PCRs, *Pj* PCR performed on blood samples may indeed be an alternative for the diagnosis of PCP. On the other hand; in the same study, none out of 26 patients with negative microscopy but positive *Pj* PCR in BAL were positive for *Pj* in serum by PCR. However, the authors did not provide any clinical data, and it remains unclear whether these patients represented cases of clinical PCP or *Pj* colonization. Furthermore, only 4% of the patients in that study were HIV-infected, and considering that non-HIV patients with PCP tend to have a lower fungal burden than HIV-infected patients with PCP [33], the results may not be relevant for HIV-infected patients. In another study on the same subject, where 98% of the included patients were HIV-infected, only 50% of the patients with positive *Pj* PCR in sputum had positive *Pj* PCR in a concomitant blood sample [9]. However, as in the aforementioned study, the authors did not make a clinical differentiation between PCP and *Pj* colonization. Moreover, 87% of the patients in that study had started empirical PCP treatment several days before blood sampling, which is likely to have contributed to the lower sensitivity reported compared to that of our study. Finally, in a very recent study, plasma samples were analysed by a *Pneumocystis* PCR assay in 80 HIV-infected patients who were diagnosed with PCP according to clinical routine. Also this study reported a lower sensitivity for blood sample-based *Pj* PCR than in our study. They found a 25% positivity-rate among patients with PCP, with a higher frequency of PCR-positivity in non-survivors than survivors [34]. However, details regarding the definition of PCP among included patients are not provided, and the authors do not report the use of any specific reference standard for PCP. Additionally, they used plasma samples for PCR analysis as opposed to our study where serum samples were used. In our pilot analysis aimed at evaluating different blood fractions for *Pj* PCR analysis, we found a high rate of PCR-inhibition when using plasma samples. This was not found for serum samples. The authors of this study do not report how inconclusive PCR results were handled, why the results are difficult to compare.

In contrast to the previous studies, the results of our study were based on a reference standard for PCP including previously defined clinical criteria. This makes it more likely that the patients representing the target disease group in our study truly suffered from PCP rather than *Pj* colonization, which may explain the higher sensitivity of *Pj* PCR in serum found in our study. Furthermore, differences in blood sample fractions, DNA extraction protocols and PCR-assays used in the different studies may also account for differences in diagnostic accuracy. Nevertheless, the number of patients included in our study was limited, why the results need to be further investigated in prospective longitudinal studies.

The specificity of the *Pj* PCR assay used in our study was 100% when performed on serum samples from blood donors, but only 71% when the control subjects consisted of HIV-infected patients; i.e. six HIV-infected negative controls had detectable *Pj* DNA in serum by PCR. It was found that seven of the HIV-infected patients selected as negative controls had respiratory symptoms; however, the retrospective nature of the study did not allow for a full evaluation of the etiology of the clinical picture, and in order to avoid the risk of exclusion bias, these patients were allowed to remain in the study. Our results showed that 5/7 of these control patients with respiratory symptoms in fact were patients with so called false positive PCR results. The possibility that some of these control patients with positive *Pj* PCR results in serum may in fact have had an undiagnosed and self-limiting PCP [35] and that the specificity of the PCR assay thus was underestimated cannot be ruled out. Another hypothetical explanation to the detection of circulating *Pj* DNA in serum in the control patients with no definite PCP may be the escape of fungal DNA, derived from *Pneumocystis* organisms colonizing the lower respiratory tract, into the circulation. We found that the median Ct values were higher, i.e. the concentrations of *Pj* DNA in the serum samples were lower in the *Pj* PCR-positive control patients than in the patients with PCP. In analogy with this, other studies have shown that *Pj* PCR Ct values in BAL samples are higher in patients with *Pj* colonization than in patients with PCP [36, 37].

We thus hypothesized that the use of a determined Ct value as cut-off for a positive *Pj* PCR-result in serum may help to discriminate true cases of PCP from patients with circulating *Pj* DNA but no manifest PCP. This proved to be true, and when using a Ct value of 34 as the determinant of a positive PCR-result in serum, the specificity increased to 90% with only a minor loss of sensitivity from 100 to 96%. Important to note, however, is that since the Ct depends on the specific PCR reaction, the Ct value of 34 proposed as a cut-off for a positive PCR result in this study may not be the most suitable cut-off for other PCR assays.

In our study, we found a sensitivity of 96% for betaglucan at a cut-off level of 80 pg/ml, which confirms the results of previous reports [21–24] showing that betaglucan in serum is a highly sensitive marker for the diagnosis of PCP in HIV-infected patients. Similar to the *Pj* PCR assay in serum, the specificity of betaglucan in serum at the lowest cut-off level of 80 pg/ml was quite low (71%) when using HIV-infected patients as control subjects. However, the combination of the two serum analyses *Pj* PCR and betaglucan with cut-off level 200 pg/ml yielded sensitivity and specificity above 90%, suggesting a very high diagnostic performance for PCP in HIV-infected patients.

It was previously shown that approximately 20% of HIV-infected patients presenting with clinical signs of lung infection have PCP [31]. With a preset pre-test probability of 20%, the negative predictive values of the *Pj* PCR or betaglucan assay in serum in our study were very high. This suggests that any of these diagnostic markers may be used to rule out PCP in an HIV-infected patient with clinical sign of lung infection.

Previous studies suggest that betaglucan levels in serum may correlate to the *Pneumocystis* fungal load in respiratory samples [19, 38] and that betaglucan levels are higher in HIV-infected patients with PCP than in those colonized by *Pj* [21]. We hypothesized that the serum betaglucan levels would correlate to the fungal burden in serum as assessed by the *Pj* PCR Ct values in the serum samples, and that Ct values and betaglucan levels might be of use to discriminate between mild and severe *Pneumocystis* disease among patients with PCP. We did find a negative correlation between betaglucan levels and Ct values in serum; however, we did not find any significant difference in median Ct values or betaglucan levels between patients with mild or severe PCP. This implies that the betaglucan level may be correlated to the fungal burden in blood; however, the fungal burden may not be correlated to the severity of *Pneumocystis* pneumonia. In analogy with these results, other authors have previously reported that the fungal burden in respiratory samples does not correlate to the severity of PCP in patients with HIV [24].

## Conclusions

In summary, this study shows that real time-PCR analysis of *Pj* in serum samples is a sensitive method with a high negative predictive value for the diagnosis of PCP in patients with HIV, and our results suggest that a negative PCR result in serum may be used to rule out the infection. Furthermore, if using a Ct value of 34 as cut-off for a positive PCR result in serum or if combining a positive *Pj* PCR result with a betaglucan level in serum > 200 pg/ml as the determinant of a positive test result, the specificity for the diagnosis of PCP is high. Although further longitudinal studies are warranted in order to draw firm conclusions, we here present a promising first assessment of serum-based microbiological analyses of *Pneumocystis jirovecii* showing that this non-invasive diagnostic approach may be an alternative method for the diagnosis of PCP in HIV-infected patients.

## Data Availability

The datasets used and/or analyzed during the current study are available from the corresponding author on reasonable request.

## Abbreviations

BAL: Bronchoalveolar lavage
Betaglucan: 1,3-β-D-glucan
CI: Confidence interval
Ct: Cycle threshold
GGO: Ground glass opacity
HIV: Human immunodeficiency virus
PCP: *Pneumocystis jirovecii* pneumonia
PCR: Polymerase chain reaction
*Pj*: *Pneumocystis jirovecii*
TMP-SMX: Trimethoprim–sulfamethoxazole
